# Effect of Charlson Comorbidity Index and Treatment Strategy on Survival of Elderly Patients After Endoscopic Submucosal Dissection for Gastric Adenocarcinoma: A Multicenter Retrospective Study

**DOI:** 10.3389/fpubh.2021.803113

**Published:** 2022-01-03

**Authors:** Wenzhe Cao, Shaohua Liu, Shasha Wang, Shengshu Wang, Yang Song, Yao He

**Affiliations:** ^1^Beijing Key Laboratory of Aging and Geriatrics, National Clinical Research Center for Geriatrics Diseases, Institute of Geriatrics, Second Medical Center of Chinese People's Liberation Army (PLA) General Hospital, PLA Medical School, Beijing, China; ^2^College of Medicine, Shaoxing University, Shaoxing, China; ^3^Division of Gastroenterology, First Medical Center of Chinese People's Liberation Army (PLA) General Hospital, Beijing, China

**Keywords:** endoscopic submucosal dissection, gastric adenocarcinoma, charlson comorbidity index, overall survival, comorbidity

## Abstract

**Background:** The optimal treatment strategy for elderly patients with early gastric adenocarcinoma (EGAC) after non-curative endoscopic submucosal dissection (ESD) remains unclear. The purpose of this research was to explore the effectiveness of additional treatments after ESD and the factors affecting survival in elderly patients (≥60 years of age) with EGAC.

**Methods:** A total of 639 elderly patients (≥60 years) treated with ESD for EGAC from 2006 to 2018 were retrospectively reviewed. Positive lymphatic infiltration, submucosal infiltration, and positive/indeterminate vertical resection margins are considered high risk factors in histology. According to the risk of lymph node metastasis in patients with EAGC and the treatment strategies adopted after ESD, patients were divided into three groups: there were 484 patients in group A with low risk, 121 patients in group B with high risk, without additional treatment, and 36 patients in group C with high risk, with additional treatment. The 5- and 8-year survival rate, as well as the prognostic factors of survival rate after ESD was studied.

**Results:** The median follow-up time was 38, 40, and 49 months, respectively. There were 3, 4, and 3 deaths related to gastric adenocarcinoma in groups A, B, and C, while deaths from other diseases were 20, 5, and 3, respectively. There were significant differences in overall survival rates between groups (94.3; 86.4; 81.2%, *p* = 0.110), but there was no significant difference in disease-specific survival rates (98.4; 92.7; 92.4%, *p* = 0.016). In the multivariate analysis, the Charlson Comorbidity Index (CCI) ≥ 2 was an independent risk factor for death after ESD (hazard ratio 2.39; 95% confidence interval 1.20–4.77; *p* = 0.014).

**Conclusions:** The strategy of ESD with no subsequent additional treatment for EGAC may be a suitable option for elderly patients at high risk, especially for CCI ≥ 2.

## Introduction

There were an estimated 16,910 new cases of early gastric adenocarcinoma and 12,720 deaths related to gastric adenocarcinoma in China in 2020 (1). The 5-year survival rate for gastric cancer is 30% (2), and its prognosis is closely related to early detection and treatment (3, 4). With the development of flexible endoscopic diagnosis and treatment tools, ESD is currently recommended as the standard surgical method for the endoscopic treatment of early gastric adenocarcinoma.

ESD has the following advantages: (1) it is less affected by the size of the lesion, and it can almost always provide adequate En bloc specimens for histological examination, so it has the greatest diagnostic and therapeutic benefits; (2) compared with traditional endoscopic mucosal resection (EMR), ESD has better oncologic outcomes.

Previous studies have showed that compared with gastrectomy, endoscopic treatment provides better surgical safety and acceptable oncology results in early gastric cancer (5–8). However, the study of comparing ESD in treating early gastric adenocarcinoma (EGAC) was lacking. Furtherly, for elderly patients with early gastric adenocarcinoma (EGAC), the best treatment strategy after ESD is still unclear, which still needs more detailed clinical research data support. Some studies proved that ESD treatment of early gastric cancer in elderly patients is even safe and feasible (9–12). However, the post-ESD treatment is currently not standardized, especially elderly patients tend to have more complications, limited life expectancy, poorer general conditions, and poor tolerance to post-ESD additional treatment. In addition, Elderly patients are at higher risk of all-cause death, which has caused people to worry and pay attention to the safety and effectiveness of additional treatments after ESD. Moreover, China's aging problem is getting more and more serious, and it is expected that many countries will face similar situations in the future. The purpose of this study was to explore the effectiveness of additional treatments after ESD and survival predictors of elderly patients (≥60 years of age) in the hospital-based EGAC cohort. It typically occurs in elderly cancer patients with multiple comorbidities and CCI is a reliable tool that can estimate prognosis of cancer based on type and number of comorbidities. Therefore, in this study, CCI was used as the survival predictor of elderly patients with EGAC.

## Methods

### Patients and Study Design

The patient population in this study came from a multicenter retrospective cohort study of five tertiary referral hospitals in China. We retrospectively reviewed the treatment and follow-up data of all elderly EGAC patients who received ESD from January 1, 2006, to December 31, 2018. The last follow-up time was December 2019.

A total of 639 consecutive elderly EGAC patients who met the following criteria were included: (i) age ≥ 60 years; (ii) treatment-naïve EGAC; (iii) pathologically confirmed adenocarcinoma of excised gastric specimen; and (iv) no metastasis. The exclusion criteria were: (i) patients with a history of surgical gastrectomy because a remnant stomach could affect survival outcomes; (ii) patients with premalignant lesions (high-grade intraepithelial neoplasia) and intraepithelial adenocarcinoma; The indication criteria for ESD in gastric cancer were: (i) no lymph node or distant metastasis was detected by computer imaging; (ii) tumor staging based on endoscopy indicating superficial invasion; and (iii) Written informed consent is required. According to the confirmed pathological results of the excised specimens, the risk of Post-ESD is classified and evaluated (low risk and high risk).

According to the resection effect and pathology, we considered the following cases to be at high risk: non-curative ESD, including positive lymphatic or/and venous infiltration, positive or indeterminate vertical margins, submucosal infiltration, and at low risk: curative resection (tumor depth does not exceed lamina propria mucosae, negative horizontal/vertical margin, and negative lymphatic and vascular invasion) or non-curative resection with tumor invasion up to MM or/and horizontal margins as positive/indeterminate. We divided patients into three groups based on post-ESD treatment strategies and the level of lymph node metastasis (LNM) risk: group A, low-risk patients; group B, high-risk patients without any post-ESD additional treatment; and group C, high-risk patients with additional treatment after ESD. Charlson Comorbidity Index (CCI) was used to assess the risk of death from comorbidities. CCI comprises 19 weighted comorbidities (such as cardiovascular disease, chronic kidney disease, uncomplicated diabetes, and liver disease, etc.) according to the original definition (13, 14). CCI quantified disease burden and comorbidity burdens, with high burden defined as a score of ≥2 (15), so the best CCI cut-off value was 2. This study was approved by the Institutional Ethics Review Committee of PLA General Hospital.

### Histological Assessment

Hematoxylin and eosin and immune-histochemical staining were performed after the specimen is cut into slices of ~2 mm, and the slices were evaluated by pathologists in each institution according to the standards for diagnosis and treatment of gastric cancer in china in 2018. We evaluated the tumor invasion depth, horizontal and vertical margin status, lymphovascular invasion, and histological characteristics based on the above standards. The depth of submucosal invasion is classified as SM1 (<500 μm) or SM2 (≥500 μm). Hematoxylin and eosin staining was used to evaluate lymphatic and venous invasions. Elastica van Gieson staining was used for the assessment of vascular invasion, and D2-40 is used for the assessment of lymphatic invasion.

### Post-ESD Management and Outcome Assessment

During the treatment of this study, doctors judged the appropriate indications for post-ESD additional treatment based on the patient's treatment, his/her own condition and personal preference for treatment strategies. The additional treatment post-ESD in our study specifically refers to gastrectomy.

The main outcome was the all-cause mortality at the end of follow-up (at least 6 months) in patients with EGAC. Secondary outcomes included the following indicators (2) the incidence of severe nonfatal adverse events and perioperative mortality; (3) additional treatment; (4) cumulative disease-specific mortality and tumor recurrence or metastasis at the end of follow-up period.

### Follow-Up

The patients were followed up at the 3rd and 6th months, and then every 6 months until the third year, and yearly afterward. Outpatient visits, blood tests, endoscopy, and computed tomography were the main follow-up methods. We defined loss of follow-up as a follow-up of fewer than 6 months with no known recurrence, metastasis, or death. The decision of post ESD additional treatment was made case by case. In general, additional treatment was recommended for all patients with positive margin cancer and ESD patients with T1b lesions, especially those with deeper, or wider invasion, lymphatic involvement, or poor differentiation. However, the decision for additional treatment also took into account age, physical condition, comorbidity, life expectancy, and, most importantly, patient preferences.

### Statistical Analysis

Statistical analyses were performed using IBM SPSS (version 26.0). Categorical statistics were represented as a number and percentage, while continuous statistics were represented as a mean average and standard deviation. Statistical methods used in this study include the Student's *t*-test (or Mann-Whitney U test), Fisher's exact test (or Pearson's chi-square test), the Kaplan-Meier method for survival analysis, and Cox hazards regression analysis. The adjustment covariates in multivariate cox regression analyses were demographic characteristics (age, sex), Post-ESD treatment strategy. As shown in **Table 3**, the category variables of sex and Post-ESD treatment strategy were used as covariates and the continuous variable of age was used as covariate. The schoenfeld test was used to evaluate the proportional hazards (PH) assumption when conducting cox regression analysis. In a sensitivity analysis, we extended the follow-up of the primary and secondary outcome to 6 months after ESD. The start of the follow-up period was defined as the initial date of the ESD treatment, while the end date of follow-up was the date of final contact or the date of death until December 2019. Cumulative survival analysis was performed with the use of Kaplan-Meier methods and curves were compared with the log-rank test. After excluding patients with follow-up <6 months, we then performed a Kaplan-Meier survival analysis. Kaplan-Meier, Cox hazards regression, sensitivity analysis were performed by R version 4.1.1. A *p* < 0.05 indicated the difference is statistically significant.

## Results

### Patients

A total of 639 elderly patients were included and analyzed, of which 527 (82.5%, 527/639) were assessed as CCI ≤ 1 group, while the other 112 (17.5%, 112/639) were assessed as CCI ≥ 2 group. Of the 527 CCI ≤ 1 patients, 100(19.0%, 100/527; group B) received ESD without additional treatment, whereas 26 (4.9%, 26/527; group C) underwent ESD with additional treatment. And, the patient details are summarized in Table 1. There were no significant differences in Post-ESD treatment strategy, sex, smoking, drinking, BMI, lesion location, tumor morphology between CCI ≤ 1 group and CCI ≥ 2 group. However, the age of CCI ≥ 2 group was higher than CCI ≤ 1 group, and there was a significant difference.

**Table 1:** Patient demographics and lesion cancer.

**Patient demographics**	**CCI ≤ 1**	**CCI ≥ 2**	***P***
	***n* = 527**	***n* = 112**	**value**
Age, years	67.0 (63.0–72.0)	69.0 (64.0–74.0)	0.003
Men	410 (77.8)	92 (82.1)	0.373
Post-ESD treatment strategy			0.247
Group A	401 (76.1)	81 (72.3)
Group B	100 (19.0)	21 (18.8)
Group C	26 (4.9)	10 (8.9)
Smoking	155 (29.4)	41 (36.6)	0.165
Drinking	140 (26.6)	38 (33.9)	0.144
BMI, kg/m2	23.8 ± 3.1	24.4 ± 3.5	0.087
Lesion location			0.672
Upper third	210 (39.8)	41 (36.6)
Middle third	124 (23.	5) 25 (22.3)
Lower third	193 (36.6)	46 (41.1)
Tumor morphology			0.979
Elevated	307 (58.3)	66 (58.9)
Flat or depressed	220 (41.7)	46 (41.1)

*Values are mean ± SD, n (%), or median (interquartile range)*.

*CCI, Charlson Comorbidity Index*.

The pathological result of ESD for elderly patients with gastric cancers was summarized in Table 2. In group B, the lesion diameter tended to be larger when compared to group C. The proportion of undifferentiated histologic appearance tended to be higher in group B than in group C (31.4 vs. 19.4%, *p* < 0.001). The ratio of positive vertical margin to positive vertical margin in group C is often higher than that in group B (33.3 vs. 9.1%, *p* < 0.001; 25.0 vs. 13.2%, *p* < 0.001; respectively). The proportion of lymphovascular invasion in group B is similarly higher than that in group C.

**Table 2:** Pathological result and outcomes of endoscopic submucosal dissection for the 639 elderly patients with gastric cancers.

**Patient demographics**	**Group A**	**Group B**	**Group C**	***P***
	***n* = 482**	***n* = 121**	***n* = 36**	
Lesion diameter, cm^a^	1.2 (0.8–2.0)	2.0 (1.2–3.0)	1.6 (1.0–2.0)	<0.001
Histologic appearance				<0.001
Differentiated	462 (95.9)	83 (68.6)	29 (80.6)	
Undifferentiated	20 (4.1)	38 (31.4)	7 (19.4)	
Depth of invasion^b^				<0.001
M	465 (96.5)	74 (61.2)	15 (41.7)	
SM1	17 (3.5)	19 (15.7)	5 (13.9)	
SM2	0	28 (23.1)	16 (44.4)	
Positive horizontal margin	0	16 (13.2)	9 (25.0)	<0.001
Positive vertical margin	0	11 (9.1)	12 (33.3)	<0.001
Lymphovascular invasion^c^	0	18 (14.9)	3 (8.3)	<0.001
Short-term clinical outcomes				
En bloc resection	480 (100.0)	84 (70.0)	25 (69.4)	<0.001
R0 resection^d^	480 (100.0)	64 (53.3)	9 (25.0)	<0.001
Hospital stay, days	13 (11–17)	13 (11–17)	17 (13–32)	<0.001
Postoperative hospital stay, days	6 (5–7)	7 (5–8)	6 (5–12)	<0.001
Hospital cost, USD	26973.6 (22301.1–32277.4)	32000.9 (25132.8–35025.8)	9389.2 (6040.5–11856.7)	<0.001
Nonfatal adverse events	15 (3.1%)	2 (1.7%)	1 (2.8%)	0.686
Postoperative bleeding	13 (2.7)	2 (1.7%)	1 (2.8%)	0.812
Delayed perforation	1 (0.2)	0	0	1.000
Pneumonia	1 (0.2)	0	0	1.000
Anastomosis stenosis	2 (0.4)	0	0	1.000
Postoperative intestinal obstruction	0	1 (0.8)	0	0.246
Adjuvant therapy				<0.001
Repeat endoscopy	0	0	5 (13.9%)	
Repeat surgery	0	0	30 (83.3%)	
Chemotherapy	0	0	1 (2.8%)	
Oncologic outcomes, follow-up^e^				
All-cause mortality	24 (5.0)	8 (6.6)	6 (16.7)	0.016
Disease-specific mortality	4 (0.8)	3 (2.5)	3 (8.3)	0.001
Recurrence/metastasis	40 (8.3)	9 (7.4)	8 (22.2)	0.015

*Values are mean ± SD, n (%), or median (interquartile range)*.

a *Naked-eye measurement of the largest-diameter lesion on stretched and nonfixed pathology specimens*.

b *Tumoral infiltration of the submucosa was subclassified as SM1 (<500 um from the muscularis mucosae) or SM2 (≥500 um from the muscularis mucosae)*.

c *Lymphovascular invasion for endoscopic submucosal dissection specimens*.

d *Horizontal and vertical margins free from cancerous and precancerous tissues (high-grade intraepithelial neoplasia)*.

e *Median 27 (range, 6–143) months*.

### Survival Analysis

**Figure 1:**
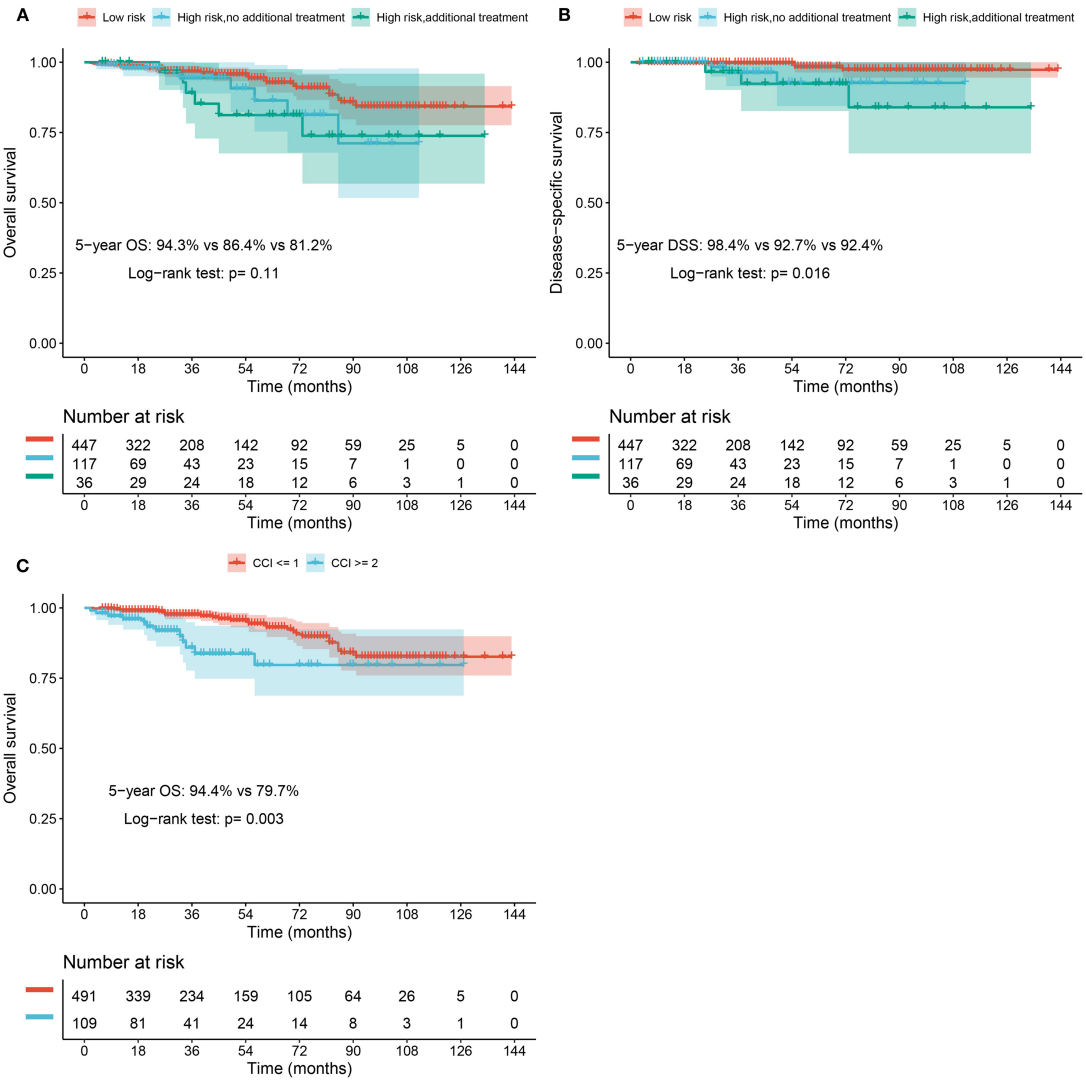
**(A)** Overall survival rate of elderly patients with EGAC after ESD stratified based on the risk of LNM. **(B)** Disease-specific survival rate of elderly patients with EGAC after ESD stratified based on the risk of LNM. **(C)** Overall survival rate of elderly patients with EGAC after ESD in CCI ≥ 2 and CCI ≤ 1 groups.

The median follow-up time of group A, group B, and group C were 38, 40, and 49 months, respectively. A total of 38 gastric adenocarcinoma patients died during the follow-up study. The 5-year overall survival rates in group A, group B, and group C were 94.3% (95% CI 94.8–98.7%), 86.4% (95% CI 75.4–99.0%), and 81.2% (95% CI 67.6–97.6%), respectively, whereas the 8-year overall survival were 84.3% (95% CI 77.6–91.5%), 71.1% (95% CI 51.7–97.8%), and 73.8% (95% CI 56.8–95.9%), respectively (Figure 1A). There was no significant difference in overall survival between the three groups (*p* = 0.11). The 5-year disease-specific survival were 98.4% (95% CI 96.4–100%), 92.7% (95% CI 84.5–100%), and 92.4% (95% CI 82.7–100%) in group A, group B, and group C, respectively (Figure 1B). The disease-specific survival between the three groups showed a significant difference (*p* = 0.016).

### Prognostic Factors for Survival

We summarized the risk factors for overall survival in Table 3. In the univariate analysis results, elder age, increased CCI, the greater risk for LNM, diabetes were significantly associated with poor overall survival. On multivariate analysis, CCI ≥ 2 (hazard ratio, 2.39; 95% CI 1.20–4.77, *p* = 0.014) was associated with impaired overall survival. We also found that age is an important independent risk factor for impaired overall survival in elderly patients (≥60 years of age) with EGAC. The 5-year overall survival rates of patients with CCI ≥ 2 were significantly lower than that of patients with a CCI ≤ 1 (79.7 and 94.4%, respectively, *p* = 0.003) (Figure 1C). According to the analysis results of Supplementary Table 1, CCI had no significant effect on overall survival (hazard ratio, 1.41; 95% CI 0.53–3.80) or disease-specific survival (hazard ratio, 2.05; 95% CI 0.21–20.10) in the low-risk group, but had a significant effect on recurrence or metastasis (hazard ratio, 2.17; 95% CI 1.10–4.28). Different cox models yielded robust results regarding significant and non-significant outcomes.

**Table 3:** Risk factors associated with poor overall survival.

**Variables**	**No. of patients**	**No. of deaths**	**Univariate**		**Multivariate**	
			**HR (95% CI)**	***p***	**HR (95% CI)**	***p***
**Post-ESD treatment strategy**
Group A	482	24	Reference		Reference	
Group B	121	8	1.78 (0.79–3.97)	0.162	2.16 (0.69–3.49)	0.290
Group C	36	6	2.26 (0.92–5.54)	0.075	2.16 (0.89–5.30)	0.094
**Risk for LNM**
Low risk	482	24	Reference			
High risk	157	14	1.96 (1.01–3.79)	0.046		
**Age, years**
Continuous variable	639	38	1.08 (1.03–1.14)	<0.001	1.08 (1.03–1.13)	0.002
CCI						
0–1	527	26	Reference		Reference	
≥2	112	12	2.74 (1.38–5.45)	0.004	2.39 (1.20–4.77)	0.014
**History of cancer**
Present	37	4	1.05 (0.56–1.99)	0.379		
Absent	602	34	Reference			
**Cardiovascular disease**
Present	285	18	1.05 (0.56–1.99)	0.871		
Absent	354	20	Reference			
**Respiratory disease**
Present	38	3	1.36 (0.42–4.43)	0.609		
Absent	601	35	Reference			
**Liver disease**
Present	40	1	0.47 (0.06–3.42)	0.456		
Absent	599	37	Reference			
**Renal disease**
Present	31	4	2.13 (0.76–6.00)	0.153		
Absent	608	34	Reference			
**Diabetes**
Present	102	13	2.78 (1.42–5.43)	0.003		
Absent	537	25	Reference			

*The p value was calculated by Cox hazards regression analysis*.

*CCI, Charlson Comorbidity Index*.

**Figure 2:**
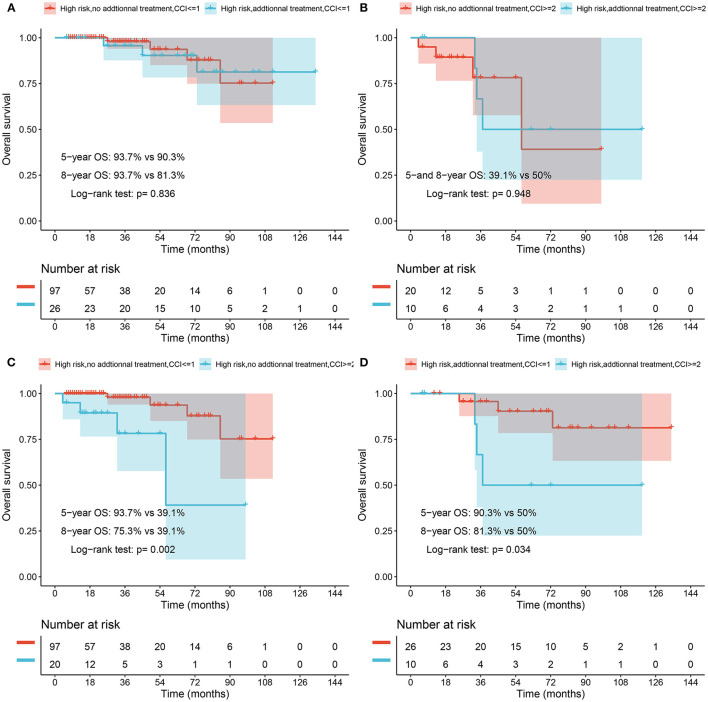
**(A)** Overall survival rate of elderly EGAC patients with high risk and CCI ≤ 1 after ESD in no additional treatment and additional treatment groups. **(B)** Overall survival rate of elderly EGAC patients with high risk and CCI ≥ 2 after ESD in the follow-up and additional treatment groups. **(C)** Overall survival rate of elderly EGAC patients with high risk and no additional treatment after ESD in CCI ≤ 1 and CCI ≥ 2 groups. **(D)** Overall survival rate of elderly EGAC patients with high risk and receiving additional treatment after ESD in CCI ≤ 1 and CCI ≥ 2 groups.

The survival rate of high-risk patients was further analyzed according to post-ESD treatment strategy and CCI classification. We re-stratified post-ESD high-risk patients (group B and group C) according to CCI classification (CCI ≤ 1 and CCI ≥ 2), and compared the overall survival as follows: each CCI scoring range was compared with and without additional treatment: CCI ≤ 1 and CCI ≥ 2 for each ESD post-processing intervention (Figures 2A,B).

Analysis of high-risk patients with CCI ≤ 1, the 5- and 8-year overall survival rates of patients who did not receive additional treatment were 93.7 and 75.3%, respectively, while those who received additional treatment were 90.3 and 81.3%, and there was no significant difference (*p* = 0.836) between the two groups receiving additional treatment and not receiving additional treatment (Figure 2C). Analysis of high-risk patients with CCI ≥ 2, the 5- and 8-year overall survival rates of the patient who did not receive other interventions were 39.1 and 39.1%, respectively, while those who received additional treatment were 50 and 50%, and those who did not receive additional treatment there was no significant difference between high-risk patients and high-risk patients receiving additional treatment (*p* = 0.948) (Figure 2D).

In high-risk cases that did not involve other interventions after ESD, patients with CCI ≥ 2 had significantly lower overall survival than those with CCI ≤ 1 (*p* = 0.002). Among the high-risk patients receiving other interventions after ESD, the overall survival rate of patients with CCI ≥ 2 was lower than that of patients with CCI ≤ 1, and the difference was significant (*p* = 0.034).

### Sensitivity Analysis

We performed a sensitivity analysis to examine the degree of bias introduced by the patients who were follow-up for <6 months. No difference was found in the distribution of treatment strategy, age, gender, comorbidities, CCI, smoking, drinking, BMI, lesion characteristics/pathology in Table 4. In view of the similar characteristics of patients, surgery, and perioperative parameters, there is no significant impact on the result analysis, despite the follow-up time being <6 months in part patients.

**Table 4:** Sensitivity analysis.

**Variable**	**Follow-Up** **≥6 Months or Death** **Before 6 Months**		***P***
	**Yes (*n* = 600)**	**No (*n* = 39)**	
Post-ESD treatment strategy			0.077
Group A	447 (74.5)	35 (89.7)	
Group B	117 (19.5)	4 (10.3)	
Group C	36 (6.0)	0	
Age, years	68.3 ± 6.3	69.6 ± 6.9	0.202
Men	476 (79.3)	26 (66.7)	0.062
**Comorbidities**			
Cardiovascular disease	273 (45.5)	12 (30.8)	0.073
Respiratory disease	38 (6.3)	0	0.105
Liver disease	39 (6.5)	1 (2.6)	0.325
Renal disease	30 (5.0)	1 (2.6)	0.493
Diabetes	97 (16.2)	5 (12.8)	0.580
Charlson comorbidity index, *n* (%)			0.095
0–1	491 (81.8)	36 (92.3)	
≥2	109 (18.2)	3 (7.7)	
Smoking	189 (31.5)	7 (17.9)	0.075
Drinking	171 (28.5)	7 (17.9)	0.154
BMI, kg/m2	23.9 ± 3.2	23.3 ± 2.5	0.213
Lesion location			0.069
Upper third	239 (39.8)	12 (30.8)	
Middle third	134 (22.3)	15 (38.5)	
Lower third	227 (37.8)	12 (30.8)	
Tumor morphology			0.354
Elevated	353 (58.8)	20 (51.3)	
Flat or depressed	247 (41.2)	19 (48.7)	
Depth of invasion			0.112
M	516 (86.0)	38 (97.4)	
SM1	40 (6.7)	1 (2.6)	
SM2	44 (7.3)	0	
Lymphovascular invasion	19 (3.2)	2 (5.1)	0.506

*Values are n (%), mean ± SD, or median (interquartile range)*.

*ESD, endoscopic submucosal dissection*.

## Discussion

In this hospital-based cohort study for elderly patients with EGAC treated using ESD, we clarify the significance of CCI as a prognostic factor. After analyzing 639 consecutive patients, our findings demonstrate the triage value of CCI regarding mortality, appropriate treatment, and survival gain after additional treatment. For patients at high-risk LNM after EGAC ESD, we suggest that for elderly patients over 60 years old with CCI ≤ 1, close observation and follow-up without additional treatment after ESD treatment may be a feasible option. For patients, the risk of LNM is very high. Additional treatment afterward is a reasonable choice.

The CCI is a reliable co-morbidity index to be used in research, especially for surgical patients. However, few previous studies aimed to explore the necessity of an additional treatment strategies for elderly patients with EGAC after ESD. In the current study, we found that there was no significant difference in overall survival rate and disease-specific survival rate between patients who received additional treatment and patients without any post-ESD treatment (16–18). Because of the shorter life expectancy of these elderly patients with high recurrence risk after surgery, the necessity of additional treatment after ESD is difficult to determine clearly, and extra treatment may not effectively prolong the life expectancy. Therefore, our research results demonstrated that CCI ≤ 1 can be regarded as a meaningful indicator for judging whether post-ESD high-risk patients aged ≥60 need adjuvant therapy after treatment. A meaningful result showed that there was a significant difference in disease-specific survival rate between patients who performed additional treatment and patients without any post-ESD treatment. Usually, compared with follow-up observations, patients with LNM identified as high-risk target population after ESD are more inclined to receive further treatment. These results indicated that some post-ESD elderly patients can benefit from additional treatment.

Several studies have clarified the relationship between CCI and complications of elderly gastric patients after ESD, which showed CCI can serve as an independent prognostic factor (12, 19–21), and the significance of CCI in patients with non-curative EGAC after ESD remained unclear. Our study demonstrated that age and higher CCI score (≥2) were independent prognostic factors in elderly patients with EGAC treated using ESD, similar to the findings reported in previous studies. Moreover, we established 2 as the optimal CCI threshold upon ROC curve analysis. According to the CCI grade and the treatment strategy after ESD, According to the CCI classification and treatment strategy after ESD, we mainly focus on the survival results of high-risk patients with EGAC after ESD, and have not explored the risk of all-cause death and primary cancer death in low-risk LNM patients with EGAC after ESD. Moreover, among elderly patients underwent ESD with high risk, we found special factors affecting high mortality, with diabetes was emphasized as a prognostic factor in univariate analysis. CCI remained an independent factor affecting survival after we eliminated the factor from multivariate analysis as it already reflected in CCI. Therefore, CCI can be used as a valuable indicator to evaluate the survival of elderly EGAC patients after ESD.

In recent studies, CCI was reported that it performed well in predicting the prognosis of various diseases, such as ischemic stroke, end-stage renal disease, cirrhosis, and lung cancer (22–24). Our findings show that among high-risk group of EGAC patients with CCI ≤ 1 or CCI ≥ 2, there was no significant difference in 5- and 8-year overall survival rates between patients who opted for additional treatment and patients who only received follow-up observation. However, the 5- and 8-year overall survival of high-risk patients with CCI ≥ 2 who received additional treatment was higher than those of patients who only received follow-up, but there was still no significant difference between the two groups. It provides a reasonable option to judge whether additional treatment is needed according to the risk of LNM and CCI score in the clinic. Therefore, we expect to apply CCI score to other advanced endoscopic procedures in elderly patients to formulate the most reasonable treatment.

The present study has several limitations. First, the sample size of this study is not large enough, especially group C, elderly patients with high-risk for EGAC after ESD often receive no additional treatment. Second, there are few studies on the effectiveness and safety of elderly EGAC patients after ESD. According to the prognosis of elderly patients with EGAC after ESD, the follow-up time required in this study is insufficient. Third, the additional treatment of patients with high risk of lymph node metastasis after ESD largely depends on the attending doctor. The indications and physical conditions of elderly EGAC patients have a greater impact on whether additional treatment is required. In addition, there are other uncertain factors such as age, family care, financial status, and patient treatment preferences (11, 25).

In conclusion, our study provides important evidence that the observation strategy without intervention after ESD for EGAC may be an acceptable or best option for elderly patients with CCI ≥ 2, as the additional treatment cannot effectively extend the life expectancy of patients. Furthermore, regardless of whether additional treatment after ESD in patients with EGAC, CCI ≤ 1 has a better survival condition than CCI ≥ 2.

## Data Availability Statement

The data analyzed in this study is subject to the following licenses/restrictions: The data sets generated and/or analyzed during the current study are not publicly available to ensure patient privacy, but are available from the corresponding author on reasonable request. Requests to access these datasets should be directed to Wenzhe Cao, caowenzhe301@163.com.

## Ethics Statement

The studies involving human participants were reviewed and approved by the Institutional Ethics Review Committee of PLA General Hospital. The patients/participants provided their written informed consent to participate in this study.

## Author Contributions

WC: conceptualization, formal analysis, methodology, software, and writing—original draft. SL: formal analysis, and writing—review and editing. ShaW: data curation. SheW: investigation and validation. YS: writing—review and editing and visualization. YH: conceptualization, funding acquisition, project administration, resources, and supervision. All authors read and approved the final manuscript.

## Funding

This study was supported by research grants from National Key Research and Development Program of China (2016YFC1303603); National Natural Science Foundation of China (81773502, 81703308, 81703285); General Research Project of Zhejiang Provincial Department of Education (Y202044677).

## Conflict of Interest

The authors declare that the research was conducted in the absence of any commercial or financial relationships that could be construed as a potential conflict of interest.

## Publisher's Note

All claims expressed in this article are solely those of the authors and do not necessarily represent those of their affiliated organizations, or those of the publisher, the editors and the reviewers. Any product that may be evaluated in this article, or claim that may be made by its manufacturer, is not guaranteed or endorsed by the publisher.
