# Supplementary material for: Effect of Charlson Comorbidity Index and Treatment Strategy on Survival of Elderly Patients After Endoscopic Submucosal Dissection for Gastric Adenocarcinoma: A Multicenter Retrospective Study
Source: Front Public Health. 2022 Jan 3;9:803113. doi: 10.3389/fpubh.2021.803113 (PMC8761651; doi:10.3389/fpubh.2021.803113)
Supplement: Supplementary file 1 [file Table_1.docx]

**Table S1.** HR(95%CI) for the association between CCI and survival outcomes among the low-risk and high-risk hospital-based population

| Variable | Low-risk hospital-based population | |  | High-risk hospital-based population | |
| --- | --- | --- | --- | --- | --- |
|  | Crude Model | Model 1 |  | Crude Model | Model 1 |
| All-cause mortality |  |  |  |  |  |
| CCI |  |  |  |  |  |
| 0–1 | Reference | Reference |  | Reference | Reference |
| ≥2 | 1.58(0.59-4.25) | 1.41 (0.53-3.80) |  | 6.00(2.08-17.33) | 4.92(1.53-15.84) |
| Disease-specific mortality |  |  |  |  |  |
| CCI |  |  |  |  |  |
| 0–1 | Reference | Reference |  | Reference | Reference |
| ≥2 | 2.19(0.23-21.19) | 2.05 (0.21-20.10) |  | 3.03(0.54-16.85) | 3.02(0.44-20.87) |
| Recurrence/metastasis |  |  |  |  |  |
| CCI |  |  |  |  |  |
| 0–1 | Reference | Reference |  | Reference | Reference |
| ≥2 | 2.17 (1.10-4.28) | 2.17 (1.10-4.28) |  | 0.56(0.13-2.46) | 0.43(0.09-1.97) |

Notes: Model 1: adjusted for age, sex. HR: hazard ratio; CI: confidence interval; CCI: Charlson Comorbidity Index
